# Clinical Management of Blood–Brain Barrier Disruptions after Active Raster-Scanned Carbon Ion Re-Radiotherapy in Patients with Recurrent Head-and-Neck Cancer

**DOI:** 10.3390/cancers11030383

**Published:** 2019-03-19

**Authors:** Thomas Held, Sati Akbaba, Kristin Lang, Semi Harrabi, Denise Bernhardt, Christian Freudlsperger, Steffen Kargus, Peter Plinkert, Stefan Rieken, Klaus Herfarth, Jürgen Debus, Sebastian Adeberg

**Affiliations:** 1 Department of Radiation Oncology, Heidelberg University Hospital, 69120 Heidelberg, Germany; thomas.held@med.uni-heidelberg.de (T.H.); sati.akbaba@med.uni-heidelberg.de (S.A.); kristin.lang@med.uni-heidelberg.de (K.L.); semi.harrabi@med.uni-heidelberg.de (S.H.); denise.bernhardt@med.uni-heidelberg.de (D.B.); stefan.rieken@med.uni-heidelberg.de (S.R.); klaus.herfarth@med.uni-heidelberg.de (K.H.); juergen.debus@med.uni-heidelberg.de (J.D.); 2 Heidelberg Institute of Radiation Oncology (HIRO), 69120 Heidelberg, Germany; 3 National Center for Tumor diseases (NCT), 69120 Heidelberg, Germany; 4 Clinical Cooperation Unit Radiation Oncology, German Cancer Research Center (DKFZ), 69120 Heidelberg, Germany; 5 Heidelberg Ion-Beam Therapy Center (HIT), 69120 Heidelberg, Germany; 6 Department of Oral and Maxillofacial Surgery, University Hospital Heidelberg, 69120 Heidelberg, Germany; christian.freudlsperger@med.uni-heidelberg.de (C.F.); steffen.kargus@med.uni-heidelberg.de (S.K.); 7 Department of Otorhinolaryngology, University of Heidelberg, 69120 Heidelberg, Germany; peter.plinkert@med.uni-heidelberg.de; 8 German Cancer Consortium (DKTK), partner site Heidelberg, DKFZ, 69120 Heidelberg, Germany

**Keywords:** brain necrosis, carbon ions, particle therapy, head and neck cancer, re-irradiation

## Abstract

*Purpose:* The aim of the current evaluation was to assess central nervous system necrosis (CNSN) after re-irradiation with carbon ions (CR) in two-hundred seventeen (*n* = 217) patients with recurrent head-and-neck cancer (HNC). *Methods:* Thirty-six (*n* = 36) patients with CNSN were assessed retrospectively regarding clinical symptoms and radiographic response. *Results:* CNSN were classified according to clinical management in line with the Common Terminology Criteria for Adverse Events (CTCAE) v5.0. At a median follow-up of 25.3 months (range 3.3–79.9 months), the median time interval until occurrence of grade I, II, and III CNSN was 9.2 months (range 2.8–75.0 months), 10.2 months (range 2.3–60.5 months), and 16.6 months (range 8.7–32.5 months), respectively. In one patient with an adenocarcinoma infiltrating the frontal lobe, an extensive CNSN grade IV was suspected but the patient declined surgical intervention. Radiographic response after treatment of CNSN grade I, II, and III, defined as ≥25% reduction of the T2 alteration on Magnetic Resonance Imaging (MRI), was observed in 4 (16.0%), 5 (29.4%), and 4 (80%) patients, respectively. *Conclusion:* CNSN occurred late and frequent after re-irradiation with carbon ions in patients with HNC infiltrating the base of skull. The clinical outcome with adequate treatment was encouraging but correct diagnosis of CNSN remains challenging.

## 1. Introduction

Improving clinical outcome while minimizing treatment-related toxicity in normal tissues embodies a major challenge of any tumor-specific treatment [1]. Re-irradiation of head-and-neck as well as brain cancer is related to an increased risk of central nervous system necrosis (CNSN) [2]. Factors contributing to treatment-related central nervous system (CNS) toxicity are among others damage to vessel structures, demyelination and generalized alterations of cytokine expression [3]. Resulting in hypoxia, these mechanisms stimulate the formation of vascular endothelial growth factor (VEGF), leading to blood–brain barrier disruption and extracellular edema [4]. Consequently, patients may experience severe neurological symptoms, e.g., headache, seizures, or cognitive dysfunction due to CNSN [1,5]. However, the exact mechanisms of CNSN formation are currently not completely understood. Further risk factors for CNSN include radiotherapy target volume, total dose, and concurrent systemic therapies [6]. The relative biological effectiveness (RBE) of carbon ions compared to photons is higher and depends on the biological system, the irradiated tissue, and the calculation model [7]. Heavy ions enable irradiation with a steep dose gradient, allowing dose escalation in the recurrent tumor while minimizing the maximum cumulative dose of normal tissue and organs at risk, e.g., the temporal lobe. However, the clinical impact of re-irradiation with carbon ions (CR) on CNSN formation is yet to be investigated. Besides radiotherapy, other treatment modalities, particularly systemic therapies, may cause CNSN [8]. The diagnosis of CNSN remains challenging since tumor progression must be considered before initiating adequate treatment. A thorough evaluation of the radiotherapy treatment plan and comparison with the high-dose area is therefore essential. Nevertheless, short-term follow-up examinations by Magnetic Resonance Imaging (MRI) imaging are frequently inevitable to distinguish locally progressive disease from CNSN.

Management of asymptomatic patients with grade I CNSN may consist of observation, regular clinical evaluations, and imaging without further treatment. Corticosteroids are utilized as a first and initial treatment choice in symptomatic patients to reduce cerebral edema and mass effects [9]. Intravenous steroid application was reported to show improved clinical results and reduced long-term side effects compared to oral intake [10]. The effectiveness of bevacizumab against CNSN was first reported in a study on patients with malignant brain tumors by Gonzalez et al. [11]. These results were substantiated by a randomized controlled trial by Levin et al., showing pronounced radiographic and clinical improvement through VEGF inhibition in patients with CNSN [12]. Eventually, Xu et al. [13] showed that bevacizumab could cause symptomatic relief and radiographic response, i.e., reduction of T2 FLAIR alteration on MRI, even with no simultaneous corticosteroid therapy. In the case of recurrent CNSN, repeated treatment with bevacizumab was reported to show similar effectiveness [14]. According to prior evaluations, a mean lesion reduction of around 50% on MRI T2-weighted sequences could be achieved by bevacizumab therapy [15,16]. Reported effects on the improvement of clinical symptoms are various, since most evaluations focused on radiographic response and treatment regimens differ. Many factors, e.g., the dose of bevacizumab, are yet to be determined. Awareness of aspects indicating CNSN, a serious complication after radiation treatment, is essential for radiation oncologists and other specialties for early and adequate clinical management.

The aim of the current evaluation was to assess the clinical and radiographic course, evaluated using MRI, and management of CNSN after re-irradiation with carbon ions in patients with recurrent head-and-neck cancer (HNC).

## 2. Results

### 2.1. Tumor Features

Most tumors were primarily located in the base of the skull (*n* = 17, 47.2%), the orbit (*n* = 5, 13.9%), and the temporal bone (*n* = 4, 11.1%), as well as the cavernous sinus (*n* = 3, 8.3%) and the nasopharynx (*n* = 3, 8.3%). The majority of tumors were adenoid cystic carcinomas (ACC, *n* = 24, 66.7%). Staging was conducted prior to CR according to the eighth edition of the Union for International Cancer Control (UICC) tumor-node-metastasis (TNM) classification. All tumors were classified as locally advanced, due to invasion of the skull base. At the start of CR, 10 patients (27.8%) showed distant metastatic spread, most commonly in the lungs (*n* = 9, 90.0%).

### 2.2. CNS Necrosis

At a median follow-up of 25.3 months (range 3.3–79.9 months), CNSN grade I, II, and III developed in 17, 13, and 5 patients, respectively (Table 1). Consequently, a total of thirty-six (*n* = 36, 16.6%) patients developed radiation-induced CNSN. In most cases CNSN were diagnosed in consensus with a radiation oncologist, neuroradiologist, and ENT-specialist in the interdisciplinary head-and-neck tumor conference. All CNSN grade III were presented and discussed in our radiooncological conference before the indication for bevacizumab therapy was confirmed. In one patient with an adenocarcinoma infiltrating the frontal lobe, an extensive, symptomatic CNSN grade IV was suspected but the patient declined surgical intervention. Regarding the short time interval of 4.5 months after CR, other differential diagnoses, e.g., abscess, seem plausible. None of the CNSN were histologically confirmed. The median time interval until occurrence of grade I, II, and III CNSN was 9.2 months (range 2.8–75.0 months), 10.2 months (range 2.3–60.5 months), and 16.6 months (range 8.7–32.5 months). The majority of CNSN were located in the temporal lobe (*n* = 30, 83.4%) or the frontal lobe (*n* = 4, 11.1%). The mean and maximum dose of CR in the respective brain area was 13.3 Gy (RBE) (range 0.2–50.2 Gy (RBE)) and 53.2 Gy (RBE) (range 3.3–64.0 Gy (RBE)). An alpha/beta of 2 Gy was utilized for brain tissue according to the Quantitative Analyses of Normal Tissue Effects in the Clinic (QUANTEC) [17].

### 2.3. Clinical Management and Radiographic Response

**Figure 1:**
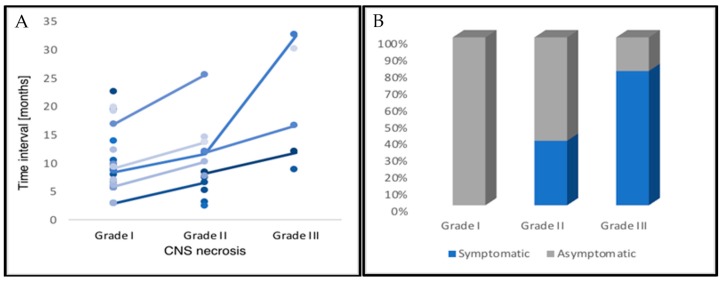
Depicted is (**A**) the clinical course of central nervous system necrosis (CNSN) after re-irradiation until occurrence on Magnetic Resonance Imaging (MRI) follow-up scans, as well as (**B**) the respective fraction of patients with symptomatic CNSN. Asymptomatic patients showed radiographic progression of CNS necrosis and therefore received treatment with corticosteroids or bevacizumab.

**Figure 2:**
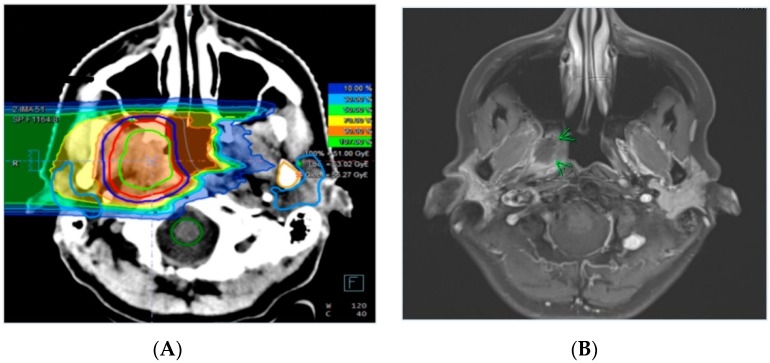
Carbon ion re-irradiation treatment plan of a patient with a recurrent adenoic cystic carcinoma (ACC) of the right nasopharyngeal wall treated with 51 Gy (RBE) in 17 fractions (**A**). Corresponding axial contrast-enhanced T1-weighted MR sequence of the same patient prior to re-irradiation (**B**).

**Figure 3:**
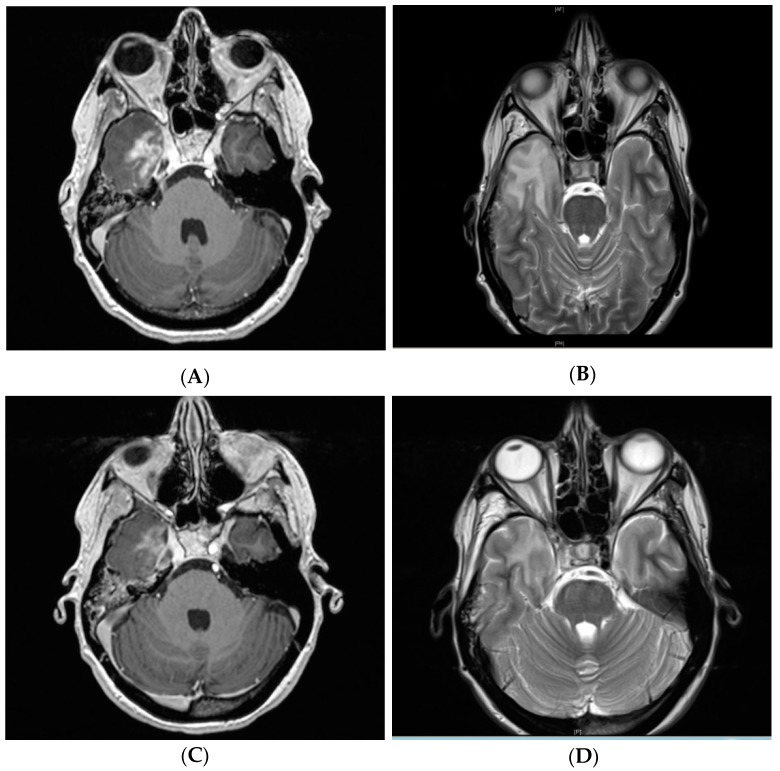
T1-weighted contrast-enhancement (**A**) and T2 alteration (**B**) post-treatment follow-up MR imaging of the same patient from Figure 2 showing grade III symptomatic CNSN in the right temporal lobe 11.9 months after treatment. Radiographic response after intravenous bevacizumab 7.5 mg/kg administered at two-week intervals for four treatments with residual T1-weighted contrast-enhancement (**C**) and T2 hyperintensity (**D**). The patient also responded clinically with swift recovery from double vision, vertigo, and headaches.

Clinical symptoms related to CNSN grade I, II, and III were observed in zero (0%), five (38.5%), and four (80%) patients, respectively (Figure 1). CNSN grade II developed from initial grade I blood–brain barrier changes in six patients (*n* = 6, 46.2%). Radiographic response after treatment of CNSN grade I, II, and III, defined as ≥25% reduction of the T2 abnormality (axial diameter) on MRI, was observed in four (16.0%), five (29.4%), and four (80%) patients. From all patients with grade III CNSN, three patients (75%) received four cycles of bevacizumab and one patient (25%) received eight cycles. In three patients (*n* = 3, 11.1%), the initial diagnostic assumption was progressive disease, but the final evidence strongly supported CNSN due to a response to dexamethasone. A clinical case of a patient with a grade III symptomatic CNSN after CR is shown in Figure 2 and Figure 3.

## 3. Discussion

The main differential diagnosis for CNSN is tumor progression, representing a diagnostic dilemma and is a common clinical problem [18]. As imaging techniques are improving rapidly, diagnostic sensitivity and specificity were increased using MRI, enhancing diagnostic accuracy. However, frequently, the severity of clinical symptoms, such as neurocognitive impairment, and the radiographic manifestations on MR imaging show no correlation. Therefore, the time interval after radiotherapy until occurrence of suspected CNSN should be integrated in the diagnostic evaluation. Mixtures of CNSN with partial recurrent tumor in biopsy specimens were reported in 11% of patients with malignant gliomas after resection and adjuvant radiation treatment [19]. Evaluation of short-term imaging follow-up and reaction to CNSN specific treatment (e.g., corticosteroids) are crucial in selected patients and can further advance the complex diagnostic process. Currently, definite diagnosis of CNSN can be achieved only via examination of surgical brain tissue specimens, although sampling errors are frequent [1]. The ability of functional imaging, particularly PET, to differentiate early tumor progression from CNSN is limited and the diagnostic contribution is therefore insignificant [20]. In the current analysis, the diagnostic assumption was incorrect in at least three patients (*n* = 3, 11.1%). Initially progressive disease was suspected but the final evidence strongly supported CNSN due to a response to dexamethasone.

In general, patients with HNC infiltrating the skull base receiving re-irradiation are at risk to develop CNSN, particularly in the temporal and frontal lobes. As reported by Hu et al. [2], 10.6% of patients that underwent re-irradiation of nasopharyngeal tumors developed CNS necrosis grade after treatment. As indicated by the mean and maximum dose of 13.3 Gy (RBE) and 53.2 Gy (RBE) in the respective brain area, maximum constraints for brain tissue [17] were not adhered to in the current evaluation. Due to tumor infiltration into the skull base, higher total doses were allowed upon receipt of informed patient consent. This might explain the elevated occurrence of CNS necrosis (*n* = 36, 16.6%) in the current analysis. However, out of all patients with CNS necrosis, only five patients developed symptomatic CNSN ≥ grade III (*n* = 5, 2.3%). The clinical impact of (re-)radiotherapy with heavy ions compared to photons on CNSN formation is currently unclear. However, compared to photons, minor anatomical changes (e.g., air-filled cavities) can already cause large uncertainties regarding treatment planning.

As early blood–brain barrier alterations are frequently transient and resolve spontaneously, observation represents a reasonable approach. Correct timing for commencement of CNSN-specific treatment is crucial and guided by the patients’ clinical symptoms. In this current analysis, the median time interval until development of blood–brain barrier changes (i.e., CNS necrosis grade I) was 9.2 months. However, CNSN occurred as late as 32.5 months after re-irradiation. According to the standard procedures in our clinic, patients with symptomatic CNSN received initial treatment with oral dexamethasone for two weeks, then reduced gradually. Clinical benefits of intravenous, highly dosed, pulsed application of corticosteroids were reported in previous assessments [13]. Intravenous bevacizumab 7.5 mg/kg was administered at two-week intervals for four treatments in the current assessment. An MRI scan was performed four weeks after the last treatment. The optimal dose of VEGF inhibition treatment is yet to be determined as previous studies reported total doses between 5 mg/kg to 30 mg/kg per treatment [16,21]. Although Xu et al. [13] could prove effectiveness of bevacizumab monotherapy, the role of simultaneous or sequential application of corticoids remains unclear. Furthermore, patients may benefit from neuropsychologic evaluations and interventions, such as occupational therapy [1].

As reported in previous studies [12,13], MRI was selected as the imaging modality for radiographic response assessment in the current analysis. Only 29.4% of patients with CNSN grade II receiving dexamethasone showed a ≥ 25% reduction in T2 abnormality. Bevacizumab could reach response rates of 80.0% in patients with grade III CNSN, in line with prior studies [12,13]. However, long-term follow-up is merited to identify CNSN recurrence. Corticosteroids might be beneficial in terms of long-term effectiveness, due to anti-inflammatory effects. Alternatively, bevacizumab, primarily affecting extracellular edema, may be repeated regularly. In this current study, one patient (*n* = 1, 25.0%) under bevacizumab therapy received two cycles (eight treatments) due to CNSN recurrence.

There are several limitations of our analysis worth mentioning. First, the evaluation was of a retrospective nature and data curation was conducted from patients’ medical records, radiological reports, and imaging. Statistical analysis was limited to descriptive statistics due to limited sample size. Diagnosis of CNSN was non-standardized in the consensus of a radiation oncologist and neuroradiologist and the brain lesion volume was not quantified. No surgical analysis of brain tissue specimens was performed as diagnosis was based on MRI signal alterations and clinical symptoms after correlation with the radiotherapy treatment plan. Although the median follow-up of 25.3 months was acceptable, there is no long-term data available in the current analysis.

A misinterpretation of CNS necrosis as tumor progression might have serious consequences for the patient (e.g., chemotherapy, operation, re-radiotherapy). Interdisciplinary discussion between radiation oncologists and other specialties is therefore crucial to create awareness of CNSN and improve clinical management of this serious treatment complication. CNSN occurred late and frequent after re-irradiation with carbon ions in patients with HNC infiltrating the base of the skull. The clinical outcome in the case of adequate treatment was encouraging but correct diagnosis of CNSN remains challenging.

## 4. Materials and Methods

### 4.1. Patient Selection

Screening was conducted utilizing the cancer register of the National Center for Tumor Diseases (NCT) after approval by the regional ethics committee of Heidelberg University. Patients with diagnosed HNC, at least one prior course of radiotherapy, and re-irradiation in-field or at the field margin [22] were considered for the retrospective assessment. Only patients with recurrent tumors with infiltration of the skull base and close proximity to the temporal and frontal lobes were included in the analysis. Patients with lymphoma, sarcoma, chordoma, and plasmocytoma were excluded. Finally, two-hundred seventeen (*n* = 217) patients with HNC that underwent CR between 2010 and 2017 at our institution were evaluated.

### 4.2. Treatment Planning and Radiotherapy

Patients were immobilized with a thermoplastic head-mask system. Computed tomography scans with a 3 mm slice thickness were utilized for treatment planning and contrast-enhanced T1-weighted magnetic resonance imaging for image registration. Treatment planning was conducted using Syngo PT Planning version 13 (Siemens^®^, Erlangen, Germany). According to standard procedures at our institution, the clinical target volume (CTV) consisted of the visible tumor on contrast-enhanced CT or MRI (gross tumor volume, GTV) with a safety margin of 2–5 mm. Depending on patient positioning and beam arrangement, a safety margin of 2–3 mm was added for the planning target volume (PTV). Treatment was performed at our institution utilizing the active raster-scanning method with daily image-guidance by orthogonal X-rays and position correction in six degrees of freedom.

The initial course of irradiation consisted of 3D-conformal RT in 52 patients (24.0%), intensity-modulated radiation therapy (IMRT) in 75 patients (34.6%), bimodal RT (IMRT and carbon ion boost) in 58 patients (26.7%), and other (e.g., brachytherapy) in 13 patients (6.0%). The radiation therapy technique was unknown in 19 patients (8.7%). Patients with bimodal RT received a median dose of 50 Gy (range 45–56 Gy) with photon IMRT and a median dose of 24 Gy (RBE) (range 18–24 Gy (RBE)) with carbon ions. The median dose of CR was 51 Gy (RBE) (range 39–60 Gy (RBE)) in 3 Gy (RBE) fractions and the median PTV of CR was 85.3 ccm (range 13.3–286.5 ccm). None of the patients received simultaneous systemic therapy during CR. The median total tumor lifetime dose after initial radiotherapy and CR was 133.9 Gy (range 112.5–152.3 Gy). An alpha/beta of 2 Gy was utilized for ACC and 10 Gy for squamous cell cancer and other tumor entities. Detailed patient and treatment characteristics are shown in Table 2.

### 4.3. Radiographic and Clinical Evaluation

All CNSN after CR were evaluated non-standardized by a radiation oncologist and neuroradiologist based on radiographic criteria on MR imaging as previously reported [12,13]. Increased signal on T2-weighted sequences and T1-weighted contrast-enhancement on MRI in the high-dose were utilized as criteria for the assessment of CNSN. In addition, CNSN were categorized prior to statistical analysis according to clinical management, in line with CTCAE v5.0 (Table 3). CNSN were classified according to clinical management in watch-and-wait (grade I), oral dexamethasone for 14 days, then reduced gradually (grade II), intravenous bevacizumab 7.5 mg/kg in two-week intervals for four treatments (grade III), and surgical intervention (grade IV). If the T2 alteration on MRI showed progression despite adequate clinical management, the cases were classified one grade higher, even if the patients did not have symptoms related to CNSN.

Follow-up included contrast-enhanced MRI scans of the head-and-neck, conducted six weeks after CR and then every three months within the first year after treatment. In addition, clinical symptoms and adverse events related to treatment were recorded as non-standardized after an assessment by a radiation oncologist during every follow-up visit.

### 4.4. Statistics

Statistical analysis was performed using SPSS Statistics 25 (IBM^®^, New York, NY, USA). Radiographic response regarding CNSN was evaluated from the end of re-irradiation to the occurrence of local failure. Adverse events related to CR were evaluated from the patients’ medical records according to the CTCAE version 5.0.

## 5. Conclusions

CNSN occurred late and frequent after re-irradiation with carbon ions in patients with HNC infiltrating the base of skull. The clinical outcome with adequate treatment was encouraging but correct diagnosis of CNSN remains challenging.

## Figures and Tables

**Table 1:** Central nervous system necrosis (*n* = 36 patients).

CNS Necrosis	Patients	%
Classification		
Grade IV	1	2.8
Grade III	5	13.9
Grade II	13	36.1
Grade I	17	47.2
Localization		
Temporal lobe	30	83.4
Frontal lobe	4	11.1
Other	2	5.5
Radiographic Response *		
Grade IV	n.a.	n.a.
Grade III	4	80.0
Grade II	5	29.4
Grade I	4	16.0
	Median	Range
Time Interval		
Grade IV	n.a.	n.a.
Grade III	16.6	8.7–32.5
Grade II	10.2	2.3–60.5
Grade I	9.2	2.8–75.0

* ≥25% reduction of the T2 alteration (axial diameter) on Magnetic Resonance Imaging (MRI).

**Table 2:** Patient and treatment characteristics (*n* = 36 patients).

Patient Characteristics	Patients	%
Female/Male	19/17	52.8/47.2
*ECOG* status		
0	25	69.4
1	11	30.6
Histology		
ACC	24	66.7
Adenocarcinoma	4	11.1
Acinar cell carcinoma	3	8.3
HNSCC	2	5.6
Other	3	8.3
Tumor Site		
Skull base	17	47.2
Orbit	5	13.9
Temporal bone	4	11.1
Cavernous sinus	3	8.3
Nasopharynx	3	8.3
Other	4	11.1
Treatment characteristics	Median	Range
Initial RT (EQD2)	70	50–81
Carbon ion re-irradiation (Gy (RBE))	51.0	39.0–60.0
PTV re-irradiation (ccm)	85.3	13.3–286.5
Cumulative Dose (EQD2)	133.9	112.5–152.3
RT-Interval (years)	4.1	0.3–46.5
Prior tumor-specific treatments	2	1–7

Abbreviations: Eastern Cooperative Oncology Group (ECOG); adenoid cystic carcinoma (ACC); head-and-neck squamous cell cancer (HNSCC); equivalent dose in 2 Gy fractions (EQD2); planning target volume (PTV); radiotherapy (RT).

**Table 3:** Clinical management of CNSN after radiotherapy based on severity of symptoms according to Common Terminology Criteria for Adverse Events (CTCAE) v5.0. Treatment is categorized into observation (grade I), highly dosed corticosteroids for 14 days, then reduced gradually (grade II), bevacizumab 7.5 mg/kg at two-week intervals for four treatments (grade III), or surgical intervention (grade IV).

Radiotherapy of Head-and-Neck or Brain Cancer			
Target volume?	Total dose?	Dose distribution?	Systemic therapy?
Regular clinical and radiographic follow-up			
No symptoms (Grade I)		Moderate (Grade II)	
Observation		Corticosteroids	
Radiographic or clinical progression			
Severe (Grade III)		Life-threatening (Grade IV)	
Bevacizumab		Surgery	
